# RISK AND PROTECTIVE FACTORS FOR ABUSE AND NEGLECT IN DAILY CAREGIVING

**DOI:** 10.1093/geroni/igz038.2003

**Published:** 2019-11-08

**Authors:** Maria Yefimova, Carolyn Pickering, Christopher Maxwell, Frank Puga, Tami Sullivan

**Affiliations:** 1 Stanford Healthcare, Palo Alto, California, United States; 2 School of Nursing, University Of Texas Health Science Center At San Antonio, San Antonio, Texas, United States; 3 School of Criminal Justice, Michigan State University, East Lancing, Michigan, United States; 4 Department of Psychiatry, School of Medicine, Yale University, New Haven, Connecticut, United States

## Abstract

The stress-process model suggests a variety of factors related to the stress-experience as important in the formation of outcomes including elder abuse and neglect (EAN). Multi-level modeling with days (n=831) nested within caregivers (N=50) was used to evaluate relationships between theoretically-based risk and protective factors and odds of EAN. Disruptions in the daily routine are a significant risk factor for abuse and neglect. Participating in a meaningful activity at least twice a day with the care recipient is a significant protective factor for neglect (OR=0.19; CI=0.06-0.64; p=0.01), but not abuse. Hypotheses that spending the full day together would increase the risk of EAN, and receipt of instrumental support and caregiver participation in self-care would decrease risk, were not supported. Findings demonstrate that the risk of EAN varies from day-to-day in the presence and absence of contextual factors. Moreover, abuse and neglect may have different etiologic pathways.

